# Field Evaluation of a Point-of-Care CD4 Analyzer for Monitoring HIV Patients in the Interior of the Amazon Region, Brazil

**DOI:** 10.1371/journal.pone.0121400

**Published:** 2015-04-23

**Authors:** Ione Conceição Pinto, Meritxell Sabidó, Analice Barbosa Pereira, Maeve B. Mello, Andrea de Melo Xavier Shimizu, Bruna Lovizutto Protti, Adele Schwartz Benzaken

**Affiliations:** 1 Fundação de Medicina Tropical Doutor Heitor Vieira Dourado (FMT-HVD), Manaus, Amazonas, Brazil; 2 TransLab, Department of Medical Sciences, Universitat de Girona, Catalunya, Spain; 3 Facultade la Salle, Manaus, Amazonas, Brasil; 4 Joint United Nations Programme on HIV/AIDS (UNAIDS), Brasilia, Brazil; 5 Departamento de DST, Aids e Hepatites Virais, Secretaria de Vigilância em Saúde, Ministério da Saúde, Brasília, Brazil; 6 Instituto Leônidas e Maria Deane, Oswaldo Cruz Foundation, Manaus, Brazil; Johns Hopkins University, UNITED STATES

## Abstract

**Objective:**

To evaluate the accuracy of the PIMA point-of-care CD4 analyzer (PIMA) under field conditions in comparison to the current CD4 count system (FACSCalibur), and to evaluate the operational suitability and acceptability of health professionals (HP) and HIV-patients in using the PIMA in health clinics in the Amazon Region.

**Methods:**

CD4 counts were measured onsite by the PIMA using fingerprick blood and in the reference laboratory by both the PIMA and FACSCalibur using venous blood. We used the Bland–Altman method to estimate the mean bias, and calculated the sensitivity and specificity at <200 and <500 cell/μL thresholds. Patients (n = 404) and HP (n = 7) were interviewed on the acceptability and operational suitability of the PIMA.

**Results:**

Using fingerprick blood (n = 337), the PIMA showed a concordance correlation coefficient (Rc) of 0.81, mean difference of -111.9 cell/μL, 93.1%/98.5% sensitivity, and 89.2%/56.7% specificity at <200 and <500 cell/μL thresholds, respectively. Venous blood (n = 340) showed an Rc of 0.89, mean difference of -83.4 cell/μL, 98.3%/97.5% sensitivity, and 93.9%/66.0% specificity at <200 and <500 cell/μL thresholds, respectively. The capillary PIMA was well accepted and found operationally appropriate by patients and HP.

**Conclusions:**

The agreement between both instruments was poor and the PIMA underestimated CD4 cell counts, which was more pronounced at CD4 counts ≥500 cell/μl. The PIMA’s performance with fingerprick blood was less reliable than its performance with venous blood. In Brazil, where antiretroviral treatment is initiated regardless of CD4 counts, the PIMA’s systematic bias towards CD4 underestimation may limit its role for monitoring HIV-patients.

## Introduction

In Brazil, access to appropriate tests to manage patients living with HIV/AIDS remains a challenge.[1] CD4^+^ T-cell counts in Brazil are determined by fluorescence-activated cell sorting (FACS) using flow cytometres, which are costly, require cold chains, need routine technical maintenance, require skilled technicians, and are primarily located in urban centres far from sample collection points.[2] In isolated areas in the interior of the Amazon, logistical and operational barriers to transporting samples and a lack of laboratory infrastructure combined with scarce technical staff represent considerable obstacles to the provision of quality care to people living with HIV/AIDS in the region.

Recently, a new technology has been developed to count CD4 cells. The new PIMA point-of-care (POC) CD4 analyzer (Alere PIMA CD4, Waltham, MA, USA) has a quick turnaround time, providing both CD3 and CD4 counts in only 20 minutes. Because of its cartridge-based system, rechargeable battery, and small size, it can be used in non-laboratory settings with little infrastructure.[3] The PIMA POC CD4 analyzer has already been evaluated in several contexts, showing similar results to the conventional techniques for measuring CD4 counts (4-colour CD3/4/8/45 BD FACSCalibur flow cytometry).[4–10] However, the PIMA analyzer showed a tendency to give a lower CD4 count in some studies.[4, 5, 8, 9, 11, 12] A systematic review of the impact of POC CD4 testing on HIV care suggested that POC testing can positively increase the proportion of patients receiving CD4 measurements and results as well as reduce the time to eligibility assessments.[13] However, evidence for increasing rates of antiretroviral treatment (ART) initiation and retention into care remains unclear.

Nevertheless, these potential benefits depend on the appropriate use and performance of the PIMA POC analyzer under field conditions that can be affected by the training of health professionals using the equipment, their skills in obtaining blood samples, the local infrastructure at the clinics, and hot and humid conditions.[14] These local characteristics may affect the accuracy of the PMIA analyzer’s CD4 counts. In this sense, this study aims to evaluate the accuracy of the PIMA POC CD4 analyzer under field conditions using two methods for blood sample collection (fingerprick versus venous blood) and compare them, as well as compare each of them to the current method of counting CD4 cells (FACSCalibur) in a reference laboratory in the city of Manaus. This study also aims to assess patient and health professionals performance and acceptability of the new PIMA POC CD4 analyzer in specialized clinics that provide care and treatment of people living with HIV (SAE, standing for *Serviço de Assistência Especializada em HIV/Aids* in Portuguese) in the interior of the Amazon region.

## Methods

### Study setting

The study was conducted between July 2013 and March 2014 in two SAE located in two municipalities of the interior of the Amazonas State (Tabatinga in Alto Solimões Region, at the triple border Brazil-Peru-Colombia, and Parintins in Médio Solimões Region), and in one SAE in Manaus. The SAE were selected based on the number of HIV patients enrolled at those locations, and the distance to the reference laboratory.[15] Before the introduction of POC CD4 testing, blood samples were collected once a week and sent to Manaus. Patients were asked to return for the results after approximately two months.

The study protocol was approved by the local Ethics Committee of Fundação de Medicina Tropical Doutor Heitor Vieira Dourado (FMT-HVD) in Manaus, the Brazilian National Committee for Ethics and Research (CONEP), and received a non-research determination by the Associate Director of Science of the Center for Global Health of the U.S. Centers for Disease Control and Prevention.

### Study population

Individuals were recruited consecutively among the HIV-positive patients who arrived at each SAE for routine follow-ups with blood collection for CD4 counts during a 6-month period. They were included if they were ≥ 18 years of age and agreed to participate demonstrated by a signed informed consent form.

Health professionals involved in routine examinations of SAE HIV patients were trained in the use of the PIMA POC CD4 analyzer. This included three pharmaceutical-biochemists (one per site) and four laboratory technicians (one in Tabatinga, one in Parintins, and two in Manaus).

### Study procedures

Four different PIMA analyzers were used throughout the study, one in Parintins, two in Tabatinga, and one in Manaus. When sample collection in Parintins and Tabatinga was completed, the four PIMA analyzers were transferred to the laboratory in Manaus. The participants provided a 25 μL finger prick capillary sample of blood that was obtained using a Sardest Safety lancet (1.5 mm with blade, 1.6 mm depth; Sarstedt AG & CO., Nümbercht, Germany) and was immediately processed on site. Approximately half of a drop of blood was directly loaded into the cartridge.[16] The results were provided to the patients 20–30 minutes later.

Each patient also provided 5 mL of venous blood collected in BD Vacutainer tubes containing K2 EDTA (Becton Dickinson, Franklin Lakes, New Jersey, USA). These samples were kept at room temperature (20 to 25°C) and shipped by boat or plane at the same temperature to the reference laboratory at FMT-HVD Manaus. Both storage and transportation temperature and humidity were recorded. After shipment to the reference laboratory, venous samples were processed using both the PIMA POC CD4 analyzer and the FACSCalibur (Becton Dickinson Biosciences, San Jose, California, USA). All testing was performed according to manufacturer’s instructions.[17] Laboratory analyses of venous blood samples were performed within 48 hours after venipuncture, which is in agreement with the instructions for the use of PIMA POC CD4. Operators of POC and laboratory instruments only had access to the results of either one or the other instrument, i.e., they were blinded to reciprocal results of the same patient sample.

When the PIMA POC CD4 analyzer generated an error code due to rejection of the sample or aborted result, a second measurement was performed. If the second analysis did not provide a result, the error was considered repetitive, and the sample was excluded from the comparison analysis.

Two months after the start of the study, the field coordinator observed health professionals performance in using the PIMA POC CD4 analyzer and interviewed them to identify factors that facilitated or were considered barriers to the performance of the test. Trained health professionals administered a questionnaire to patients who collected demographic data, information on access to the SAE, opinions on the reference CD4 count test, knowledge of and experience with the new POC CD4 analyzer, and comparative opinions of both tests.

### Quality control

Internal quality control was performed daily for both the PIMA POC CD4 analyzer and the BD FACSCalibur CD4 according to manufacturers’ instructions.[16, 18]. During the study period, only once the PIMA analyser, located in Parintins, did not produce consistent results for normal control samples.

For external quality assessment (EQA), the Ministry of Health supported an Inter-laboratory Quality Control Program. As one of the National CD4/CD8 Network laboratories, the reference laboratory in Manaus received proficiency testing panels for the EQA of its FACSCalibur. For the EQA of the PIMA CD4 analyzer, proficiency testing panels were created at the reference laboratory in Manaus and distributed to the sites. Onsite results were evaluated for their accuracy in comparison to known specimens.

### Data analysis

The data were analysed using MedCalc version 14.12.0 (MedCalc Software, Mariakerke, Belgium) and Stata 10.0 software (Statacorp, College Station, TX, USA). Descriptive analyses were used to summarize patient characteristics and the acceptability of the PIMA to both patients and health professionals. Paired t-tests were performed as appropriate to compare the differences in means.

We assessed the agreement between the paired test results from the PIMA and FACSCalibur using Lin’s concordance correlation coefficient, Rc.[19] Poor agreement was defined as an Rc <0.90, moderate as an Rc of 0.90–0.95, substantial as an Rc of 0.95–0.99, and almost perfect as an Rc >0.99.[20] This coefficient includes a measurement of precision (Pearson’s correlation, ρ), and accuracy (bias correction factor, C_b_).[19] Bland-Altman [21] and Pollock [22] analyses were performed to calculate the mean bias and limits of agreement (LOA), which are the mean bias ± 1.96 standard deviation (SD) of all the paired measurements. We calculated the relative bias ([PIMA CD4 testing—FACSCalibur]/mean), which is better suited than the mean absolute bias to compare absolute CD4 counts between two methods. The PIMA POC analyzer using capillary blood or venous blood and the FACSCalibur were also compared using Passing-Bablok regression analysis, which is a non-parametric linear regression and less sensitive to the presence of outliers.[23] The results were compared across SAEs and by CD4 cell count ranges. The accuracy of the PIMA POC analyzer was assessed through sensitivity, specificity, and positive and negative predictive values at clinically relevant thresholds of <200 and <500 cells/μl and was compared with reference testing on the FACSCalibur. These cut off points were selected because, according to Brazilian guidelines, they represent the initiation of chemoprophylaxis for *Pneumocystis jirovecii (CD4 counts* <200 cells/μl) and at the time of the study, the initiation of triple antiretroviral treatment *(CD4 counts* <500 cells/μl).[24]

## Results

A total of 408 subjects were recruited for the study, of which five did not accept any testing and were excluded from the accuracy analysis. In total, 403 subjects had at least one test performed and were included in the accuracy analysis (174 from Manaus, 96 from Parintins, and 133 from Tabatinga). Of the 403 patients, one did not accept fingerprick, therefore 402 tests were performed on PIMA using blood collected through fingerprick (PIMA capillary blood test). Of these, 32 tests (12.6%) generated an error instead of a valid result. These samples were retested, obtaining 10 repetitive errors (2.5%). Regarding samples collected through venous blood, of the 403 samples collected, 349 were analysed using PIMA venous blood test as, 34 samples were not transported to the reference laboratory, 15 samples arrived at the reference laboratory after 48 hours and were discarded, and in five, the test was not performed at the reference laboratory. Of the 349 venous samples, nine test (2.6%) generated an error instead of a valid result in the PIMA analyzer. These samples were retested, obtaining six repetitive errors (1.7%). In 12 blood samples, FACSCalibur was not performed. Therefore, testing results were available for 392 PIMA capillary blood test, for 343 PIMA venous blood test, and for 342 FACSCalibur. In total, 337 capillary samples and 340 venous samples were analysed with both the PIMA with FACSCalibur.

### PIMA performance versus FACSCalibur

The mean capillaryPIMA POC CD4 analyzer result was 363.3 (range: 16–1668), and the corresponding mean CD4 count using FACSCalibur was 478.2 (range: 12–1990) (Table 1). The mean venous PIMA CD4 result was 391.8 cells/μl (range: 18–1416), and the corresponding mean on FACSCalibur was 475.2 cells/μl (range: 12–1990). The overall mean CD4 for both capillary and venous blood samples in the PIMA CD4 analyzer was significantly lower than the mean CD4 count using the FACSCalibur (p<0.001).

**Table 1:** Comparison between CD4+ T-cell counts produced on paired patient samples by the PIMA POC CD4 analyzer and by conventional FACSCalibur testing, by PIMA POC CD4 testing.

Comparison	PIMA capillary vs. FACSCalibur (venous)	PIMA venous vs. FACSCalibur (venous)
N	337	340
CD4 cell/μl PIMA (mean, range)	366.3 (16–1668)	391.8 (18–1416)
CD4 cell/μl FACSCalibur (mean, range)	478.2 (12–1990)	475.2 (12–1990)
Paired t-test for difference in means (95% CI; p)	<0.001	<0.001
Concordance correlation coefficient, Rc (mean, 95% CI)	0.81 (0.78–0.84)	0.89 (0.87–0.91)
Pearson’s correlation, ρ	0.91	0.95
Bias correction factor C_b_	0.89	0.93
Absolute (cell/μl) bias* and LOA	-111.9 (-352.9, 129.1)	-83.4 (-269.0, 102.3)
Relative (%) bias and LOA	-26.5 (-88.0, 35.0)	18.6 (-56.2, 19.0)

*Bland-Altman analysis of bias (average difference)

LOA: limits of agreement (lower, upper)

**Fig 1:**
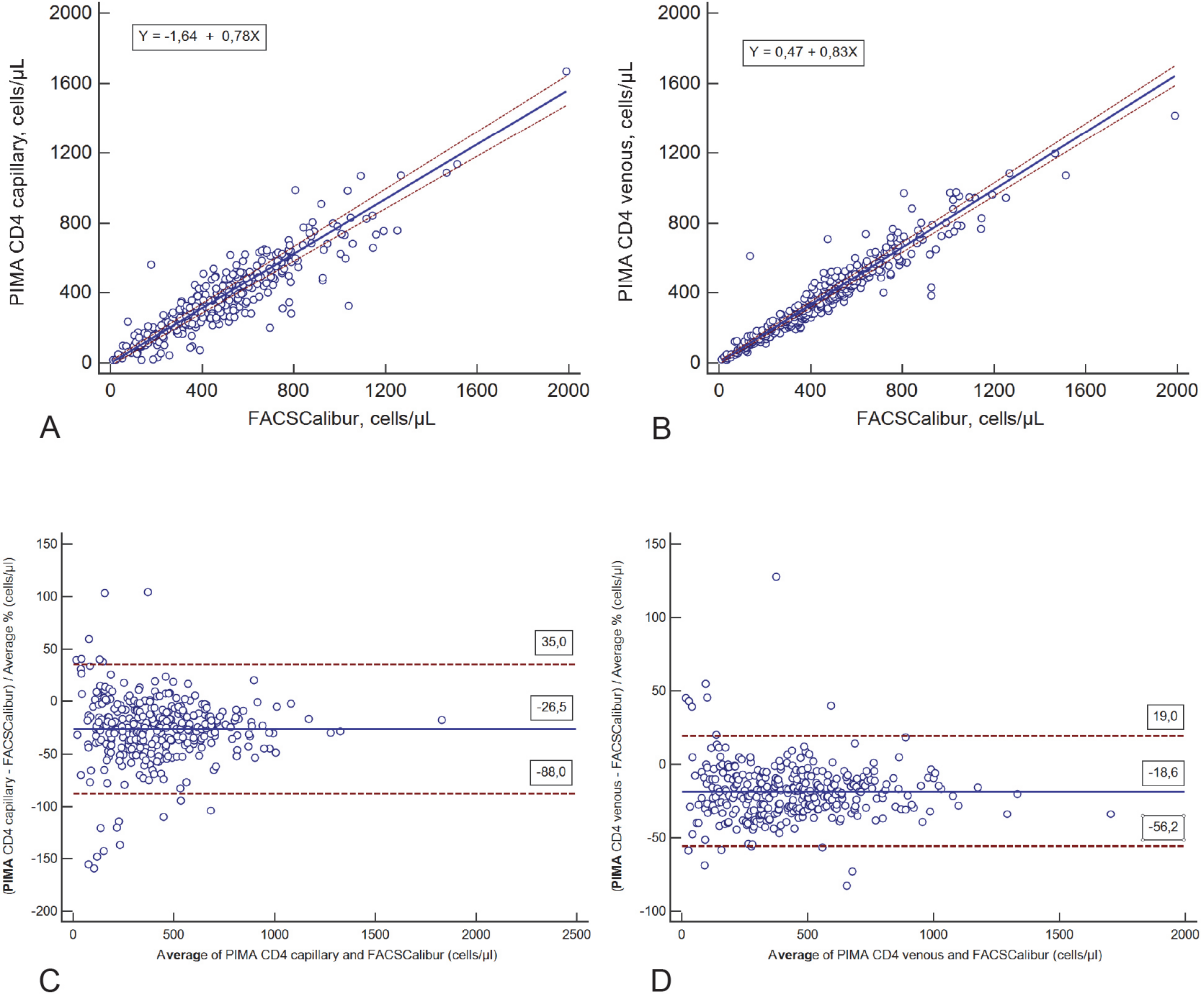
Comparison between the PIMA POC CD4 analyzer using capillary blood or venous blood samples and FACSCalibur. Passing-Bablok regression plots: comparison of absolute CD4 counts obtained by the PIMA POC CD4 analyzer using capillary blood samples (A) and venous blood samples (B) versus FACSCalibur using venous blood samples. The solid blue line represents the regression line and the dashed lines represent the 95% CI for the regression line. The corresponding graphs the relative bias between the PIMA POC CD4 analyzer and the FACSCalibur are represented in Pollock plots for capillary blood samples (C) and venous blood samples (D). The solid blue line represent the mean bias. The dashed lines represent the upper and lower limits of agreement (LOA = mean bias ± 1.96 *SD)*.

PIMA POC CD4 cell count results showed poor concordance with the FACSCalibur instrument for both methods of blood sample collection (Rc 0.81 for fingerprick capillary blood samples and Rc 0.89 for venous blood samples) (Table 1). Passing-Bablok regression plots showed slopes of 0.78 (95% CI: 0.75 to 0.82) for fingerpirck capillary blood samples and 0.83 (95% CI: 0.80 to 0.85) for venous blood samples (Fig 1).

PIMA POC CD4 cell counts using fingerprick capillary blood samples produced absolute CD4 counts with an absolute bias of -111.9 cells/μl (LOA: -352.9, 129.1) and with a relative bias of -26.5% (LOA: -88.0, 35.0) when compared with venous blood samples tested on a laboratory FACSCalibur instrument (Table 1). The PIMA CD4 analyzer using venous blood presented an absolute bias of -83.4 cells/μl (LOA: -269.0, 102.3) and a relative bias of 18.6% (LOA: -56.2, 19.0) when compared with venous blood samples run in the FACSCalibur instrument (Table 1). When data were stratified by CD4 cell count ranges, results for both of these comparisons showed that the absolute mean bias increased with higher CD4+ T cell counts (Table 2).

**Table 2:** Comparison between CD4+ T-cell counts produced with paired patient samples by the PIMA POC CD4 analyzer and by conventional FACSCalibur testing, by CD4 cell count.

COMPARISON	<200	200–499	≥500
PIMA capillary vs. FACSCalibur (venous)			
N	84	170	83
PIMA CD4 cell/μl (mean, range)	117.7 (16–198)	338.53 (201–499)	674.75 (502–1668)
FACSCalibur CD4 cell/μl (mean, range)	169.3 (12–390)	465.26 (75–1038)	817.30 (177–1990)
Paired t-test for difference in means (95% CI; p)	<0.001	<0.001	<0.001
Absolute (cell/μl) bias* and LOA	-51.6 (-192.0, 88.9)	-126.7 (-361.5, 108.1)	-142.5 (-433.6, 148.5)
Relative (%) bias and LOA	-31.0 (-120.3–58.3)	-28.9 (-79.6, 21.7)	-17.0 (-57.7, 23.8)
**PIMA venous vs. FACSCalibur (venous)**			
N	76	166	98
PIMA CD4 cell/μl (mean, range)	122.20 (18–199)	342.87 (200–499)	683.92 (501–1416)
FACSCalibur CD4 cell/μl (mean, range)	148.99 (12–354)	430.14 (210–926)	804.58 (135–1990)
Paired t-test for difference in means (95% CI; p)	<0.001	<0.001	<0.001
Absolute (cell/μl) bias* and LOA	-26.8 (-98.0, 44.4)	-87.3 (-232.6, 58.1)	-120.7 (-376.5, 135.1)
Relative (%) bias and LOA	-16.8 (-65.8, 32.2)	-21.8 (-50.9, 7.4)	-14.6 (-53.3, 24.1)

*Bland-Altman analysis of bias (average difference)

LOA: limits of agreement (lower, upper)

However, the mean percent bias was higher for samples with lower CD4 counts (200–499 cells/μl) (capillary: -28.9%, venous: -21.8%) than for the higher CD4 stratum at > 500 cell/μl (capillary: -17.0%, venous: -14.6%) (Table 2). Table 1 shows that concordance correlations between the PIMA CD4 analyzer and FACSCalibur results were lower with blood samples collected by fingerprick than with venous blood in the PIMA analyzer. Fingerprick blood samples resulted in slightly lower CD4 cell counts than venous blood samples in the PIMA.

We also compared performances between different study sites (Table 3). The mean CD4+ T-cell counts by the PIMA using capillary blood samples were similar between sites (Manaus: 362.6 cells/μl; Parintins: 372.7 cells/μl; Tabatinga: 368.5 cells/μl). However, as shown by the concordance correlation coefficients and LOA bias in Table 3, the performance of the PIMA CD4 test was better in Manaus, were the reference laboratory is located, than in Tabatinga and Parintins.

**Table 3:** Comparison between CD4+ T-cell counts produced with paired patient samples by the PIMA POC analyzer and by conventional FACSCalibur testing, by municipality.

COMPARISON	MANAUS	TABATINGA	PARINTINS
PIMA capillary vs. FACSCalibur (venous)			
N	173	98	66
PIMA CD4 cell/μl (mean, range)	362.6 (16–1668)	368.5 (58–1087)	372.7 (17–1138)
FACSCalibur CD4 cell/μl (mean, range)	436.3 (12–1990)	510.4 (75–1466)	540.2 (52–1512)
Paired t-test for difference in means (95% CI; p)	<0.001	<0.001	<0.001
Concordance correlation coefficient, Rc (mean, 95% CI)	0.90 (0.88–0.93)	0.69 (0.60–0.77)	0.77 (0.69–0.85)
Pearson’s correlation, ρ	0.96	0.85	0.92
Bias correction factor C_b_	0.94	0.81	0.84
Absolute (cell/μl) bias* and LOA	-73.7 (-247.9, 100.6)	-141.8 (-430.3, 146.6)	-167.5 (-419.6, 84.7)
Relative (%) bias and LOA	-15.8 (-56.6, 24.9)	-31.9 (-99.4,35.5)	-46.4 (-120.5, 27.7)
**PIMA venous vs. FACSCalibur (venous)**			
N	173	97	70
PIMA CD4 cell/μl (mean, range)	374.4 (18–1416)	402.8 (57–1198)	419.8 (32–1084)
FACSCalibur CD4 cell/μl (mean, range)	436.3 (12–1990)	507.1 (75–1466)	527.3 (52–1512)
Paired t-test for difference in means (95% CI; p)	<0.001	<0.001	<0.001
Concordance correlation coefficient, Rc (mean, 95% CI)	0.93	0.85	0.85
Pearson’s correlation, ρ	0.97	0.95	0.94
Bias correction factor C_b_	0.96	0.90	0.91
Absolute (cell/μl) bias* and LOA	-61.9 (-211.8, 88.1)	-104.3 (-292.7, 84.0)	-107.4 (-341.5, 126.7)
Relative (%) bias and LOA	-14.1 (-47.5, 19.4)	-25.0 (-58.1, 8.1)	-20.8 (-67.5, 25.9)

*Bland-Altman analysis of bias (average difference)

LOA: limits of agreement (lower, upper)

### Accuracy of the PIMA versus the FACSCalibur

The diagnostic accuracy of the PIMA POC CD4 measurements was compared to that of the FACSCalibur at <200 cells/μl (Table 4). The PIMA POC CD4 capillary blood sensitivity was 93.1% (95% CI: 83.3–98.1%) and the specificity was 89.2% (95% CI: 85.0–92.6%). The PIMA POC CD4 venous blood sensitivity was 98.3% (95% CI: 91.1–100%) and the specificity was 93.9% (95% CI: 90.5–96.4%). Increasing the threshold to 500 cell/μl increased the sensitivity and the positive predictive value (PPV) of the PIMA capillary blood test (98.5% and 76.0%). For the PIMA venous blood test, sensitivity slightly decreased at a threshold of 500 cell/μl (97.5%), although the PPV increased (80.2%). However, the specificity and negative predictive value declined for both tests.

**Table 4:** Sensitivity, Specificity, NPV, and PPV of capillary PIMA analyzer results versus FACSCalibur at 200 CD4+ T-cell/μl and 500 cell/μl thresholds.

FACSCalibur vs. PIMA	< 200 cell/μl		< 500 cell/μl	
	PIMA capillary	PIMA venous	PIMA capillary	PIMA venous
Sensitivity (95% CI), n/N	93.1% (83.3–98.1%) 54/58	98.3% (91.1–100%) 59/60	98.5% (95.6–99.7%) 193/196	97.5% (94.2–99.2%) 194/199
Specificity (95% CI), n/N	89.2% (85–92.6%) 249/279	93.9% (90.5–96.4%) 263/280	56.7% (48.1–65.0%) 80/141	66.0% (57.5–73.7%) 93/141
PPV (95% CI), n/N	64.3% (53.1–74.4%) 54/84	77.6% (66.6–86.4%) 59/76	76.0% (70.2–81.1%) 193/254	80.2% (74.6–85.0%) 194/242
NPV (95% CI), n/N	98.4% (96–99.6%) 249/253	99.6% (97.9–100%) 263/264	96.4% (89.8–99.2%) 80/83	94.9% (88.5–98.3%) 93/98

### PIMA acceptability and operational suitability

We interviewed 404 patients (280 males, 69.3%) with a mean age of 35.9 years (range: 18–62). Mean time for patients to reach the health clinic was 2.4 hours (range: 0–72). Almost one-fifth (19.3%, 78/404) reported difficulties in getting to the clinic; and among them, 47.4% (36/76) patients reported lack of or delays in transportation used to reach the clinic. Almost one-half (44.6%, 179/404) reported having incurred transportation costs in coming to the clinic. Most (88.9%, 359/404) have had prior CD4 counts using conventional test (FACSCalibur), and approximately half (53.2%, 213/400) reported that the delivery of results was unsatisfactory. In contrast, 97.5% (387/397) agreed that the time to obtain results with the PIMA was short and satisfactory. Almost half (47.4%, 186/392) trusted FACSCalibur results the most, although 78.6% (313/398) of patients stated that they also trusted the PIMA results. Most (65.3%, 258/395) found venipuncture more painful than a fingerprick, although 20% (44/220) reported that the fingerprick caused discomfort. Most (67.1%, 265/395) of the respondents stated that given the choice they would choose PIMA to count their CD4+ T-cells.

All health professionals (100.0%, 7/7) found the PIMA easy to use and results easy to interpret, and valued the short amount of time to provide test results to patients (85.7%, 6/7). Most (85.7%, 6/7) found the instrument suitable for daily use in the SAE. Almost half (42.9% 3/7) thought that the instrument presented error messages often, but most (71.4%, 5/7) could easily identify them. Most thought that the PIMA could easily replace the reference test (71.4%, 5/7) and trusted the PIMA results (71.4% 5/7). In contrast, 42.9% (3/7) health professionals believed that the PIMA results were less reliable than the FACSCalibur, and only 42.9% (3/7) would recommend the use of the PIMA instead of the reference test. Regarding using fingerprick to collect blood for the PIMA, health professionals perceived the method easy to perform (85.7%, 6/7), less painful for the patient (85.7%, 6/7), and able to provide results as reliable as through venipuncture (85.7%, 6/7).

Observations of the execution of the PIMA showed inadequate fingerprick collection (insufficient sample and squeezing) and/or cartridge use during internal quality control (damage to cartridges during operation and air bubbles in samples) in Parintins. Temperature and humidity was only adequately monitored in Manaus.

## Discussion

In the context of the Amazon, with hot and humid climate and remote settings, PIMA measurements using venous blood samples and particularly using fingerprick blood samples were in poor agreement with results of the FACSCalibur test. The PIMA analyzer tended to underestimate CD4 cell counts. Most of the studies comparing the PIMA CD4 analyzer (using capillary or venous specimens) to FACSCalibur have found similar underestimations by the PIMA.[4, 5, 8–12] However, we documented a larger negative bias from the PIMA placed at the two remote sites that was greater than 100 cell/μl for capillary blood. This divergence might be related with less experienced health professionals at those sites and/or conditions of sample shipment. We cannot exclude the possibility that weather conditions could have played a role in these divergent results. Temperature and humidity were monitored systematically for all shipments of samples from Tabatinga field site to the reference laboratory in Manaus, but only in one out of seven shipments from Parintins. To further explore the influence of temperature and humidity during shipment on study results we run a sensitivity analysis. The PIMA CD4 results using venous blood without the un-monitored shipment (n = 290 venous samples) showed a similar concordance correlation coefficient (Rc 0.90) but a lower bias (-77.8 cells/μl [LOA: -246.2 to 90.7]) than that obtained with the overall 340 venous samples shipped. Therefore, conditions during shipment might have played a role in the poorer results for the PIMA in remote sites of the Amazon.

The negative bias of the PIMA analyzer was more pronounced for CD4 counts ≥ 500 cell/μl than at lower CD4 ranges. Similar divergences have been observed in previous comparisons between the PIMA CD4 Analyzer and FACSCalibur, with greater underestimations by the PIMA at higher CD4 counts (>500 cell/μl).[4, 5] The PIMA performed better using venous blood than fingerprick blood samples. Although this has also been observed in previous studies,[10] the greater bias and larger LOA reported with fingerprick blood could be a matter of concern and might be related to fingerprick sampling method. In fact, although most of the health professionals considered fingerprick collection easy to perform, the study coordinator in one of the SAEs observed inadequate squeezing. This result suggests that additional training and regular monitoring are essential to maintaining the ongoing performance of the PIMA POC CD4 analyzer, as demonstrated for other POC testing.[25] The PIMA’s sensitivity improved using capillary blood samples at a higher CD4 threshold of 500 cell/μl, rather than of 200 cell/μl threshold, although the performance decreased slightly for when venous blood samples were used. This finding is consistent with two previous studies from Kenya[26] and Uganda[7] in which sensitivity of the PIMA increased when the analysis was performed using a CD4 cut-off of 500 cells/μl. Again, the PIMA results obtained from fingerprick blood samples were less precise than of those using venous blood samples.

In spite of the poor performance of the PIMA POC CD4 analyzer under the study conditions, we believe our findings to have clinical applicability. Brazil has recently adopted universal ART initiation to all HIV positive patients regardless of their CD4 cell counts. Under the Test & Treat approach, the rapid assessment of CD4 cell counts became less critical for ART initiation, but it is still an important tool for clinical decision on the adoption of prophylaxis of co-infections, particularly in remote areas such as those studied here. However, if the PIMA POC CD4 analyzer is to be used to monitor CD4 cell counts in HIV positive patients over time, this systematic underestimation may warrant further attention. On the other hand, many ART programs in resource-limited settings still rely on immunologic and/or clinical presentation to indicate ART initiation, to measure responses to therapy, and to determine when to change to a second-line regimen.[27] In the context of the use of the PIMA POC CD4 analyzer for determining eligibility for ART, underestimation of CD4 cells is not a major concern given that it would imply in earlier treatment initiation for some patients.

From an operational perspective, 32 additional PIMA capillary tests were required to provide CD4 results on 402 patients, resulting in an error rate of 12.6%. This error rate is consistent with previous evaluations of PIMA capillary testing, in which error rate ranged from 10% to 20%.[5, 8, 10, 11, 28, 29] Ten patients did not receive PIMA capillary tests result on site due to repeated machine errors. The PIMA analyzer using venous blood showed a lower rate of errors of 2.6% (9/349) than the PIMA analyzer using capillary blood. The most common errors may partially be explained by inadequate or inappropriate sample collection or cartridge use. The error rate of the PIMA capillary testing reported may influence the perception of patients and health professionals on the reliability of the tests results. Moreover, the high error rate negatively influences the time needed to deliver a test result. In an economic evaluation of a mobile program that used PIMA POC capillary testing, when an error rate of 3% was included in the analysis, the total test per cost increased slightly from $23.76 to $24.49.[30] With regards to operational aspects, the PIMA POC CD4 analyzer was well accepted by patients and health professionals, although health professionals showed low trust in the PIMA analyzer results, which could affect their buy-in if the equipment was adopted for patient management. In our study, health professionals considered fingerprick easy to perform but inadequate fingerprick collection was observed. The fact that blood samples can be collected by fingerprick using lancets is another important operational advantage in areas of limited infrastructure. Nevertheless, it is crucial to reinforce training to avoid underperformance of the equipment.

Immediate on-site testing is especially important in a region characterized by long distances and where transportation is a barrier to reaching clinics for one out of five patients. At those remote sites, at least two visits to the health clinic is required before results were available. The PIMA POC CD4 analyzer could allow patients to receive their CD4 results earlier and immediately begin treatment, thereby reducing one of the largest causes of HIV-related mortality, particularly in those areas were a high proportion of people are diagnosed very late stages of AIDS. The PIMA POC CD4 analyzer is suitable for small clinics that run up to 25 test daily, as those studied here, given that it take approximately 20 minutes to read and print one sample result. [5] This makes the PIMA POC CD4 analyzer suitable for the context of the Amazon region characterized by small and isolated villages and limited laboratory capacity.

The study presents limitations. There might be inter-observer variations in equipment handling given that several technicians were involved in the study. We did not assess the repeatability of the PIMA results. A previous study showed that duplicate POC CD4 cell counts with fingerprick blood produced results with repeatability similar to that of the FACSCalibur using venous blood.[4] Manabe *et al*. performed duplicate measurements on both capillary and venous samples using two different PIMA POC CD4 analyzers. Precision of the duplicate testing showed a significant difference in the PIMA capillary duplicates (mean difference 33.38 cell/mL, p = 0.005), but acceptable for the PIMA venous duplicates (mean difference -6.54 cell/mL, p = 0.09).[29] Wade et al. evaluated precision by running one venous blood sample 10 times on five different PIMA instruments.[28] The Pima POC CD4 analyzer showed an intra-assay variation with mean percent coefficient of variation equal or larger than the recommended value of 10% (or 15% in low CD4 counts).[31] Therefore, further studies on precision are required. The clinics used in this study are typical of the health centres found in the Amazon region in terms of numbers of patients and staff, infra-structures, and logistical barriers to access the clinic, and to the shipment of biological samples.

In conclusion, our data suggest poor agreement between the PIMA POC CD4 analyzer and the laboratory-based FACSCalibur, but additional studies are needed to understand the role that POC testing can play as a tool for monitoring CD4 cell counts. The implementation of the PIMA POC CD4 analyzer might empower SAEs in the management of their own patients and bring the clinical laboratory closer to the patient. Nevertheless, the performance with fingerprick samples was not as good as with venous samples, increasing the complexity of evaluating CD4 counts in areas with non-specialized health professionals. A high-quality management program with proper training needs to be implemented in parallel to guarantee the utility of the PIMA POC CD4 analyzer.
